# Prediction of Titanium Implant Success by Analysis of microRNA Expression in Peri-Implant Tissue. A 5-Year Follow-Up Study

**DOI:** 10.3390/jcm8060888

**Published:** 2019-06-21

**Authors:** Maria Menini, Paolo Pesce, Domenico Baldi, Gabriela Coronel Vargas, Paolo Pera, Alberto Izzotti

**Affiliations:** 1 Division of Implant Prosthodontics, Department of Surgical Sciences, University of Genoa, 16132 Genoa, Italy; maria.menini@unige.it (M.M.); baldi.domenico@unige.it (D.B.); paolopera@unige.it (P.P.); 2 Department of Health Sciences, University of Genoa, 16126 Genoa, Italy; gabrielafernanda.coronelvargas@edu.unige.it (G.C.V.); izzotti@unige.it (A.I.); 3 IRCCS Ospedale Policlinico San Martino, 16132 Genoa, Italy

**Keywords:** dental implants, micro-RNA, microarray, predictive biomarker, epigenomics

## Abstract

The aim of the present study is to evaluate the expression of microRNA (miRNA) in peri-implant soft tissue and to correlate epigenetic information with the clinical outcomes of the implants up to the five-year follow-up. Seven patients have been rehabilitated with fixed screw-retained bridges each supported by implants. Peri-implant bone resorption and soft tissue health parameters have been recorded over time with a five-year follow-up. Mini-invasive samples of soft peri-implant tissue have been taken three months after implant insertion. miRNA have been extracted from cells of the soft tissue samples to evaluate gene-expression at the implant sites by microarray analysis. The epigenomic data obtained by microarray technology has been statistically analyzed by dedicated software and compared with measured clinical parameters. Specific miRNA expression profiles predictive of specific clinical outcomes were found. In particular, some specific miRNA signatures appeared to be “protective” from bone resorption despite the presence of plaque accumulation. miRNA may be predictors of dental implant clinical outcomes and may be used as biomarkers for diagnostic and prognostic purposes in the field of implant dentistry.

## 1. Introduction

Currently dental implants are widely used for fixed rehabilitation of partially or completely edentulous patients and demonstrate predictable outcomes. However, the biological mechanisms of possible complications and implant failure are not clear and are debated in the dental scientific community.

In particular, the specific endogenous characteristic of the host (i.e., individual susceptibility) may strongly affect the success of the rehabilitation. Modern innovative technologies using molecular biomarkers may help in identifying individual susceptibility.

In a previous paper [1] we provided evidence that microRNA (miRNA) expression in peri-implant tissue reflects the pathological processes occurring in peri-implant tissues. 

miRNAs are small noncoding RNAs (ncRNAs) of approximately 22 nucleotides responsible for specific regulation of gene expression in a post-transcriptional manner. They are the main regulator of gene transcription and bear relevance in predicting clinical outcomes. Indeed, only less than 5% of expressed genes producing messenger RNA is really translated into proteins while microRNAs are fully functionally active in cell cytoplasm [2]. They have an important role in several biological processes, such as development, cell proliferation, apoptosis and carcinogenesis [3,4].

A major problem is currently represented by the lack of a predictive marker to personalize risk after peri-implant surgery. Indeed, some authors have suggested peri-implant soft tissue biotype as a risk indicator of peri-implants tissue disease [5,6]. However, this biomarker is rough and does not reflect the multiple pathogenic mechanisms occurring in the peri-implant tissue hampering or favoring the outcome of implant surgery. Conversely the accurate classification of patients in high or low-risk categories is fundamental to set up follow-up procedures and therapies according to the real risk of each subject. This is a pivotal step in the era of personalized medicine.

Molecular biomarkers are already extensively used in medicine to accurately classify each patient according to their real risk of developing complications. This approach, referred to as personalized or “theranostic” medicine, has been already well developed in oncology but is still under development in dental science. 

Molecular predictive biomarkers should reflect the pathogenic process modulating clinical outcome in the target tissue of the diseases. Furthermore, the molecular alteration investigated may occur years before the appearance of the related clinical consequence thus opening the possibility of preventing adverse clinical outcomes before their onset.

At this regard, miRNAs are currently identified as predictive biomarkers for degenerative diseases, because they do not undergo a post-transcriptional selection, being themselves the controllers of gene transcription. Consequently, compared to genomic or transcriptomic biomarkers, miRNA expression has by far a higher probability of being related with clinical variables representing a new tool for predictive medicine.

On the other side, several factors, including the implant surface [7], might affect dental implant success possibly through miRNA expression. Our research group demonstrated in vitro that osteoblasts change their gene expression profile according to the type of implant surface in contact with them [8]. 

The role of miRNA in implant dentistry has been established. In a clinical trial, we demonstrated that miRNA expressed by peri-implant tissues are related with the clinical outcome [2]. However, it remains to be established the long-term predictivity of miRNA analysis in a long-term follow-up study.

The aim of the present study is to examine the predictivity of miRNA profiling to categorize patients according to risk categories of developing more or less probably adverse consequences of oral implants on a long-term basis.

In particular, the expression of micro-RNA (miRNA) in peri-implant soft tissue will be correlated with the clinical outcomes of dental implants recorded up to the five-year follow-up.

## 2. Experimental Section

Between January 2013 and July 2014, 7 patients (4 women, 3 men; mean age: 64.6, range: 52–76) who were referred to the Division of Implant and Prosthetic Dentistry of the Surgical Sciences Department (DISC) of the University of Genoa (Genoa, Italy) and that required the insertion of two implants into an edentulous area were recruited if they met the following criteria: desire to be treated with fixed prostheses supported by dental implants and good general health without any contraindications for undergoing oral surgery and the related prosthodontic protocols.

Exclusion criteria included: an uncontrolled medical condition such as diabetes mellitus, immune suppression, intravenous bisphosphonate medication, oro-facial cancer, chemotherapy or head and neck radiotherapy, infarct during the preceding 6 months, heavy smokers (≥20 cigarettes/day). 

Healing time since tooth extraction in the intended implant sites had to be longer than 1 year. Post-extractive implants and implant sites that required a bone graft were excluded.

The present report was conducted in accordance with the Helsinki Declaration and was approved by the local Scientific Ethical Committee of the University of Genoa. 

Patients were instructed in detail on the planned clinical procedure and signed an informed consent before being scheduled in the experimental protocol.

Each patient of the sample received two adjacent implants: Osseotite implants (Biomet 3i, Palmbeach Gardens, FL) with a dual acid-etched (DAE) surface in the apical portion and a machined coronal part (hybrid implants), and Full Osseotite implants (Biomet 3i) completely DAE (DAE implants).

All the implants had identical macro- and micro-structure, external hexagon connection, a 4 mm diameter, a 10–13 mm length (depending on available bone). 

Implants were placed with a single-stage delayed loading approach and were assigned specific codes for blinding. 

The healing abutments remained in situ up to the 4-month follow-up appointment when impressions of the implants were taken to fabricate the fixed prostheses. No bone nor soft tissue grafting procedures were performed at the implant sites. 

Patients were accurately instructed on the hygienic procedures to be followed and were prescribed adequate dietary guidelines in order to ensure optimal recovery.

**Figure 1:**
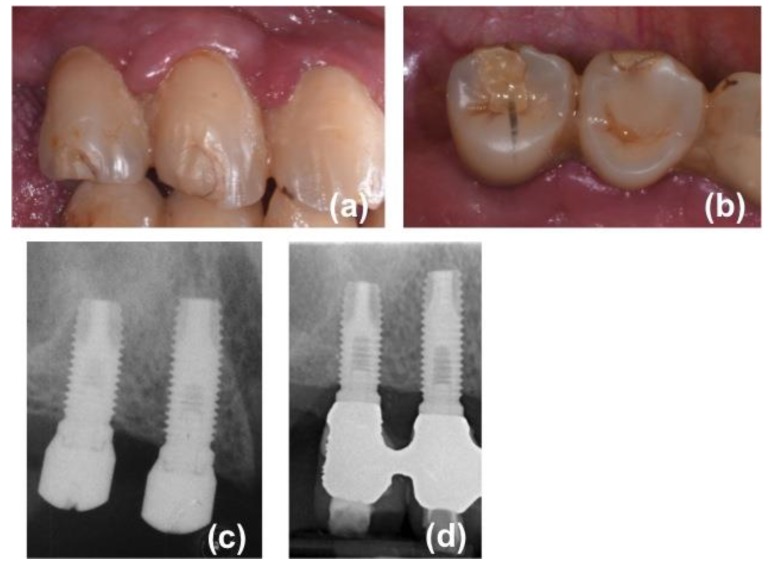
One of the patients included in the present research: (**a**) intraoral view of the bridge at the five-year follow-up appointment; (**b**) occlusal view of the bridge at the five-year follow-up appointment; (**c**) endoral radiograph three months after implant insertion; (**d**) endoral radiograph taken at the five-year follow-up appointment.

Screw-retained prostheses, provided with a metal framework and composite resin veneering material, were delivered 5–6 months (mean 24 weeks) after surgery (Figure 1). 

Spontaneous bleeding, probing depth (PD), bleeding on probing (BoP), plaque index (PI), and suppuration were recorded at 3 and 6 weeks and at 3-, 6- and 12-months post-surgery and then at the five-year follow-up appointment. At the five-year appointment the fixed prostheses were unscrewed in order to record the parodontal parameters. PD was recorded in four points for each implant (mesial, distal, buccal and lingual) using a non-metallic probe. BoP was simultaneously evaluated in four points for each implant (values from 0 to 4 for each implant). PI was measured at four points for each implant using an erythrosine gel (values from 0 to 4 for each implant).

Technical and biological complications were also recorded.

Intraoral radiographs were taken at the time of implant placement and at 3-, 6- and 12-months post-surgery and then at the five-year follow-up appointment. The implant–abutment interface was used as the reference point for interproximal bone level measurements over time. Bone resorption (BR) over the duration of the study was assessed from these reference points to the most coronal bone at the mesial and distal aspects of each implant.

The above-mentioned clinical parameters recorded at the five-year follow-up appointment were statistically analyzed and differences between DAE and hybrid implants were evaluated by Student’s *t*-test.

After 3 months from surgery, a sample of soft tissue containing both epithelium and connective tissue was collected: a mini-invasive sample of soft tissue (diameter < 3 mm) surrounding each implant was taken using a surgical blade to be histologically analyzed by miRNA microarray at the Department of Health Sciences (DISSAL) of Genoa University.

MicroRNA microarray analysis is an innovative technology, which allows the evaluations of thousands of genes in a single experiment. MicroRNAs (miRNA) have been extracted from cells of the soft tissue samples to evaluate gene-expression at the implant sites by microarray analysis.

miRNA expression data was evaluated in the peri-implant tissue as previously reported [1]. The expression of 1928 human miRNA was determined by using a commercially available microarray (miRCURY LNATM, Exiqon, Vedbaek, Denmark). 

The results of miRNA microarray have been compared with clinical parameters measured over time for five years since miRNA analysis. The previously published paper reported correlation between miRNA data and one-year outcomes (T0). The present paper reports the correlation between miRNA data and clinical outcomes recorded at the five-year follow-up appointment (T1).

All clinical continuous variables recorded at the five-year follow-up have been turned into categorical ones depending on the following criteria arbitrarily chosen by the authors:

- mean bone resorption (BR): normal/increased (normal ≤ 1 mm, increased > 1 mm)
- mean probing depth (PD): normal/increased (normal ≤ 3 mm, increased > 3 mm)
- mean plaque index (PI): low/high (low ≤ 1, high > 1)
- mean bleeding on probing (BOP): yes/no (yes ≥ 0.5, no < 0.5)

On the base of these parameters, several categories of implants (i.e., implants with normal BR vs. implants with increased BR) have been determined. 

In addition, implants with the contemporary presence of BOP, altered PD and bone resorption were considered affected by peri-implantitis (PITI).

Implant sites were also divided in two categories according to the periodontal biotype: thin (TH) and thick (TK). To evaluate the biotype, a periodontal probe was placed into the facial aspect of the peri-implant mucosa. The peri-implant biotype was categorized as thin if the outline of the underlying periodontal probe could be seen through the gingiva and thick if the probe could not be seen [9].

Relationship between miRNA and clinical events were tested by Genespring software (Agilent technologies, Palo Alto, CA, USA). 

## 3. Results

During the five-year follow-up, there were no dropouts and all the patients attended the five-year recall appointment. No implant failure occurred resulting in an implant cumulative survival rate (CSR) of 100%. No technical complications were detected, resulting in a prosthodontic CSR of 100%. All the patients anecdotally reported to be satisfied with their implant rehabilitation.

Five patients presented a thin periodontal biotype and two a thick periodontal biotype.

Mean bone resorption at the five-year follow-up appointment was 1.98 mm (min: 0.7, max: 4 mm): 1.8 mm and 2.2 mm for implants with a machined coronal portion and DAE implants respectively. 

No patient presented spontaneous bleeding or suppuration. 

No statistically significant differences were found between hybrid implants and DAE implants for the clinical variables recorded at the five-year follow-up. 

Mean and median values of the other soft tissue health parameters are reported in Table 1. 

**Figure 2:**
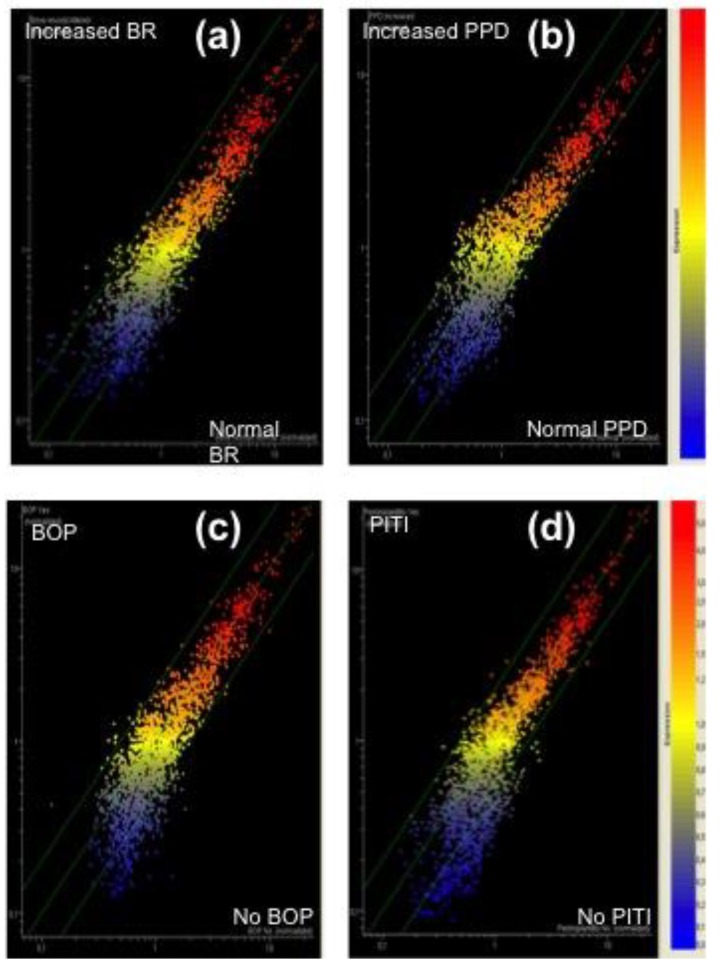
Scatter plot analyses reporting the alteration of microRNA (miRNA) profile as related to the outcome of the following clinical variables recorded at the five-year follow-up appointment: (**a**) bone resorption (BR), (**b**) peri-implant probing depth (PPD), (**c**) bleeding on probing (BOP), (**d**) peri-implantits (PITI).

**Figure 3:**
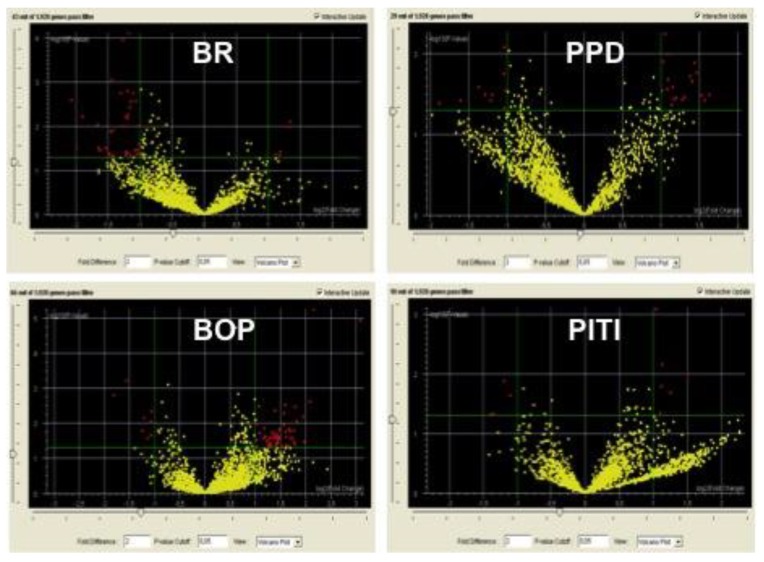
Volcano plot analyses reporting the number of significantly (<2-fold, *p* < 0.05) altered miRNAs as related to the outcome of the following clinical variables recorded at the five-year follow-up appointment: BR, PPD, BOP, PITI.

An overall view of the miRNA alterations as related to clinical outcomes occurring in the five years after surgery was performed using scatter plot (Figure 2) and volcano plot analyses (Figure 3). 

For each clinical variable, the predictor miRNAs were identified by volcano plot analysis (Table 2).

BR: 48 miRNA were significantly (Figure 3) dysregulated at T0 in patients with altered BR (altered) as compared to those with normal BR (normal) in the five years following implant surgery. Venn diagram analyses indicated that in these patients among the miRNAs altered at T0, four were predictors of BR in the following five years. These miRNAs are miR-33, miR-134, miR-200 and miR-378 (Table 2). They play a regulatory role in cell proliferation, stem cell recruitment and differentiation, osteogenesis and inflammation.

**Figure 4:**
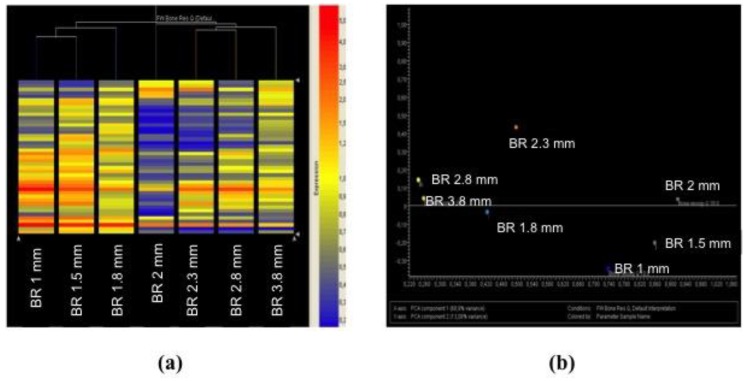
Hierarchical cluster (**a**) and principal component analysis of variance (**b**) analyses showing the occurrence of miRNA alteration in implants with BR > 2 mm.

Hierarchical cluster analyses were used to classify patients according to their miRNA expression profile as related to BR in the five years following implant surgery. Implants with a normal miRNA expression level (yellow, red) are located in the left part of the cluster, while implants with a decreased miRNA expression (blue) are located in the right part of the cluster. A severe alteration of miRNA expression at three months occurs in patients with BR ≥ 2 mm at the five-year follow-up appointment (Figure 4a). These findings were confirmed by unsupervised principal component analysis of variance (PCA) of miRNA expression profile for each implant. Indeed, all implants that had a BR ≥ 2 mm in the five years after surgery were located in a different quadrant as compared to other implants (Figure 4b).

PD: 29 miRNA were significantly (Figure 3, volcano plot) altered at T0 in patients with PD increase (increased) as compared to those having normal PD (normal) in the five years following implant surgery. Venn diagram analyses indicated that among the 10 miRNA altered in these implants at T0 (see data reported in Menini et al. [1] first year after surgery) five were predictors of PD increase in the following five years. These miRNAs are miR-30, miR-197, miR-218, miR-548 and miR-613 (Table 2). They play a regulatory role in the following biological functions: osteogenesis and cartilage homeostasis.

BOP: 66 miRNA were significantly (Figure 3, volcano plot) altered at T0 in patients with BOP (yes) as compared to those without BOP (no) at five years following implant surgery. Venn diagram analyses indicated that among the six miRNA altered in these patients at T0 (see data reported in Menini et al. [1] first year after surgery) three were predictors of BOP increase in the following five years. These miRNAs are let-7f, miR-425 and miR-548 (Table 2). They play a regulatory role in the following biological function: inflammation.

PITI: 10 miRNA were significantly (Figure 3, volcano plot) altered at T0 in patients having PITI (yes) as compared to those not having PITI (no) at five years following implant surgery. This condition was not evaluated at T0 (at the one-year follow-up appointment only two implants in one single patient presented PITI). These miRNAs are miR-517, miR-525, miR-624, miR-3128, miR-3658, miR-3692, miR-3912, miR-3920, miR-4683 and miR-4690 (Table 2). They play a regulatory role in the following biological functions: inflammation, cellular proliferation and cartilage homeostasis. 

The number of altered miRNAs as related to BR, peri-implant probing depth (PD) and BOP recorded at the five-year follow-up was dramatically increased (T1) as compared to T0 (Table 3). These findings reflect the progressive recruitment of new pathogenic mechanisms during the progression of the diseases. 

**Figure 5:**
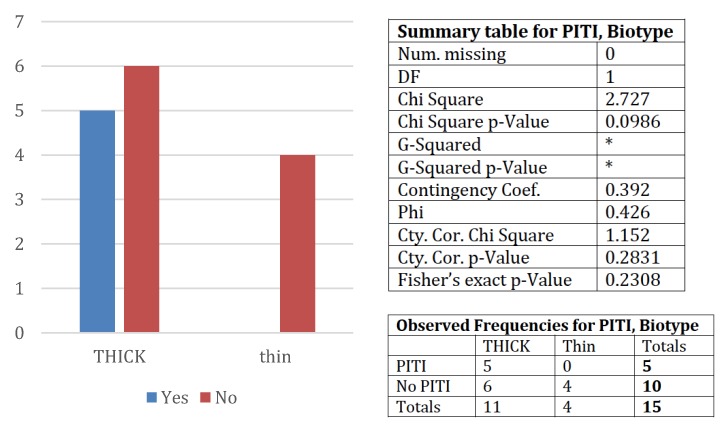
PITI occurrence as related to parodontal biotype.

**Figure 6:**
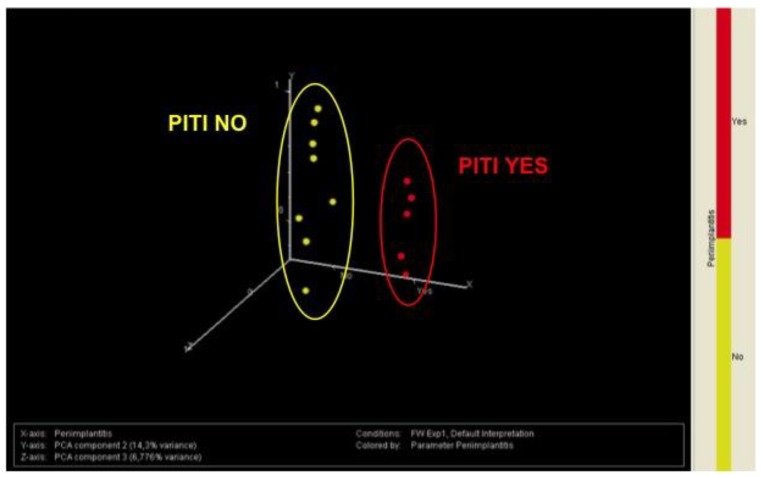
Principal component analysis of variance showing differences in miRNA expression in implants with or without PITI.

The efficacy of miRNA as biomarkers predicting clinical outcome was compared with those of periodontal biotype classified for each patient as thick (low-risk) or thin (high-risk). The ability of periodontal biotype to predict the clinical outcome of dental implants in the following five years was tested by chi square analysis (Figure 5). Of the patients with a thick biotype 33% underwent PITI, while none of the patients with a thin biotype underwent PITI. These differences were not significantly different (chi square = 2.727; chi square *p*-value = 0.0986; Fischer’s exact *p*-value = 0.2308). Conversely, miRNA expression as focused on the cluster of PITI miRNA predictors (see Table 2 and Figure 6) was able to predict PITI occurrence in all (100%) patients. 

In fact, PCA of miRNA expression at T0 (Figure 6) distinguished between patients that would develop PITI in the following five years (red dots) from those who would not develop PITI (yellow dots) in a clear-cut manner, as highlighted by the lack of any overlap between the grouping circles. The ability of miRNA profiling in predicting PITI was also statistically evaluated by k-nearest neighbors and support vector machine algorithms. The sensitivity (percentage of patients with prediction) was 80% and 93% respectively; the specificity (percentage of patients with true prediction) was 100% and 93% respectively. Accordingly, the accuracy of this test was >90% independently of the algorithm adopted.

**Figure 7:**
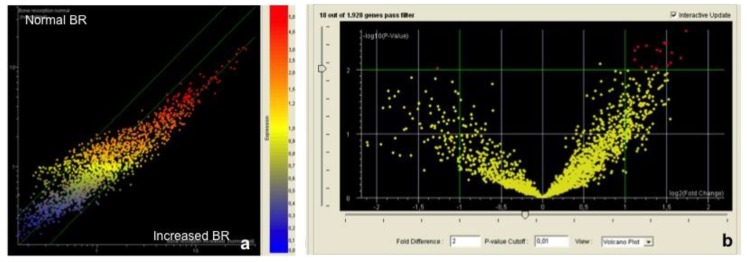
Scatter plot (**a**) and volcano plot (**b**) analyses reporting the alteration of miRNA profile as related to BR at the level of implants with high plaque index (PI).

The miRNA pattern was compared in patients undergoing plaque accumulation (high PI) between those with or without BR. The aim of this approach was to identify miRNA protecting peri-implant tissue from the adverse effect of plaque accumulation. Obtained results are reported in Figure 7. 

Both scatter plot and volcano plot identified differences between these two groups of patients. The miRNAs that protected peri-implant tissue from BR despite the presence of plaque deposits are reported in Table 4. 

## 4. Discussion

miRNA are biomarkers of several diseases and in recent years, some studies have begun to focus on the regulatory role of miRNAs in the inflammatory response [10,11].

miRNA are fundamental in controlling cell function and also influence osteoinductive pathways and inflammation in several ways. On the other side, miRNA expression is influenced by many factors and some authors have suggested that different implant surfaces may condition miRNA expression [12,13]. 

However, studies investigating the role of miRNA in implant dentistry are still scarce. Some in vitro studies are present [12,14] and studies focused on the differences of miRNA profiles between periimplantitis and periodontitis [15]. 

A recent review [16] found only one clinical trial evaluating the role of miRNA expression in the osseointegration of dental implants. This study was the investigation conducted by the present team of authors [1] and the present paper reports follow-up data for the same patients’ samples.

Our previous study suggested that miRNAs play an important role in conditioning clinical outcomes after implant insertion [1]. Specific miRNA profiles appeared to indicate lower susceptibility to peri-implant tissue inflammation and bone resorption. These findings highlighted the remarkable biological importance of miRNA expression in peri-implant tissue in determining the clinical outcome of dental implant rehabilitations. 

The present study with a five-year follow-up confirmed the potential role of miRNA in patients’ susceptibility to peri-implant disease and the potential role of miRNAs as predictors of clinical outcomes of dental implants. In fact, miRNA expression at three months of healing was predictive of the clinical variables recorded at five years of follow-up.

The miRNA mostly involved in the prediction of clinical outcomes was miR-548. Indeed, this miRNA predicted PD and BOP (Table 2). This miRNA is an established regulator of the balance between cell proliferation and apoptosis. This miRNA was found to be upregulated in patients undergoing these adverse events; indeed this upregulation results in cell proliferation arrest and apoptosis activation, two conditions fundamental in triggering degenerative diseases of the peri-implant tissue and to hamper wound healing.

The definition and etiology of peri-implant disease are controversial [17]. In particular, a clear link between plaque accumulation and bone loss has yet to be demonstrated [18,19]. The possible etiological effect of plaque accumulation on peri-implant disease may be decreased or enhanced by specific miRNAs. 

Indeed, a specific miRNA expression profile was found in patients with normal BR at five years, despite the presence of plaque accumulation ( 6,7 ), and miRNA expression analysis was far more accurate than periodontal biotype in predicting PITI occurrence in the five years after implant surgery ( 5,6 ). 

The present investigation shed light on possible biomarkers that may be predictable of dental implants clinical outcomes. The variables that can compromise implant therapy are numerous (systematic diseases, residual bone density and 3-D morphology, smoking, bruxism, regularity and effectiveness of the follow-up etc.) and the miRNA can be considered a useful tool for only evaluating the “predisposition” of the peri-implant tissues to a stable osseointegration process. 

The integrated findings derived from clinical and laboratory analysis helped with understanding the biological mechanisms affecting peri-implant tissue healing and maintenance. This will help clinicians in the choice of a better treatment plan for a predictable success of dental implant rehabilitations. In fact, specific miRNA expression profiles are predictive of specific clinical outcomes in implant dentistry and may be used as diagnostic and prognostic tools, thus affecting therapeutic strategies. These findings can be tools for a more personalized medicine approach in the treatment of edentulous patients using dental implants.

Moreover, the possible identification of “protective” miRNA also discloses the possibility of using miRNAs in future studies as therapeutic agents in implant dentistry. miRNAs might be used as possible drugs or coatings for implant surfaces, in order to improve healing and maintenance of peri-implant tissues.

## 5. Conclusions

The results of the present clinical trial suggest that miRNA may be predictors of dental implants clinical outcomes and may be used as biomarkers for diagnostic and prognostic purposes in the field of implant dentistry. 

## Figures and Tables

**Table 1:** Bone level and peri-implant health parameters recorded at the five-year follow-up appointment.

	Interproximal Bone Resorption (mm)	Plaque Index	Bleeding on Probing	Probing Depth (mm)
Mean	1.98	2.9	2.1	3.7
Median	2	3	2	4
Min	0.7	0	0	1
Max	4	4	4	6.5
P for DAE vs. hybrid implants	0.1466	0.8641	0.5817	0.6215

**Table 2:** Identity of miRNAs predictor of five-year clinical outcomes of dental implants.

Clinical Variable Predicted	miRNA
Bone Resorption (BR)	miR-33, miR-134, miR-200, miR-378
Bleeding on probing (BOP)	let-7f miR-425, miR-548
Peri-implant pocket depth (PPD)	miR-30, miR-197, miR-218, miR-548, miR-613
Perimplantitis (PITI)	miR-517, miR-525, miR-624, miR-3128, miR-3658, miR-3692, miR-3912, miR-3920, miR-4683, miR-4690

**Table 3:** Number of altered miRNAs as related to BR, PD and BOP recorded at the one-year (T0) and at the five-year follow-up (T1).

	BR	PD	BOP
**T0**	7	10	6
**T1**	48	29	66

**Table 4:** miRNA that were found to be “protective” from bone resorption despite the presence of plaque deposits (high PI) and their fold variation. The fold change indicates the ratio of miRNA expression intensity between the two categories of implants stratified according to differences in bone resorption (increased vs. normal) next to implants with high PI.

Gene Name	Fold Change
hsa-miR-4677-5p	3.328
hsa-miR-3914	3.189
hsa-miR-4679	2.948
hsa-miR-378b	2.929
hsa-miR-4434	2.858
hsa-miR-32-3p/mmu-miR-32-3p/rno-miR-32-3p	2.804
hsa-miR-1/mmu-miR-1a-3p	2.769
hsa-miR-4778-5p	2.681
hsa-miR-99b-3p/mmu-miR-99b-3p/rno-miR-99b-3p	2.615
hsa-miR-7-5p/mmu-miR-7a-5p/rno-miR-7a-5p	2.496
hsa-miR-3146	2.389
hsa-miR-4439	2.296
hsa-miR-539-3p	2.247
hsa-miR-222-3p/mmu-miR-222-3p/rno-miR-222-3p	2.225
hsa-miR-124-5p/mmu-miR-124-5p/rno-miR-124-5p	2.169
hsa-miR-4689	2.167
